# In-between duty and hope for recognition, the experience of physiotherapists working in a university hospital during the COVID-19 first wave in Switzerland: a qualitative study based on focus groups

**DOI:** 10.1186/s40945-023-00169-2

**Published:** 2023-08-17

**Authors:** Claude Pichonnaz, Rose-Anna Foley

**Affiliations:** 1 Department of Orthopaedic Surgery and Traumatology, Lausanne University Hospital and University of Lausanne, Lausanne, Switzerland; 2 HESAV School of Health Sciences, HES-SO University of Applied Sciences and Arts Western Switzerland, Lausanne, Switzerland

**Keywords:** COVID-19, Disease outbreaks, Physical therapy, Focus groups, Interview, Interpretative approach, Qualitative research

## Abstract

**Background:**

Learning more about the physiotherapists’ experience, perceived role and perception of events during the COVID-19 crisis, as well as their recovery and projection into the post-crisis future, may be useful to inform stakeholders about the impact of the crisis.

The objective of this study was to investigate the experience of physiotherapists working in a university hospital in Switzerland during the 1^st^ wave of the COVID-19 crisis, more specifically their subjective experience, professional involvement, perception of management and perceived implications for the future.

**Methods:**

This interpretative qualitative study investigated the subjective experience of a purposeful sample of 12 physiotherapists using two 2 h semi-directive focus group interviews conducted by a physiotherapist in June 2020. Data were recorded, transcribed, and analysed using a thematic analysis approach. The report was approved by participants and the study was audited by a health anthropologist.

**Results:**

The most impressive points were the unprecedented nature of the crisis, the health threat, the hospital's capacity to reorganise on a large scale and the solidarity between colleagues. Participants expressed a high level of commitment to their role despite the potentially serious repercussions at an individual level. Pride and stress coexisted for those directly involved in the crisis, while those working in a reduced activity department felt anxious and idle. The need for immediacy in decision-making and action led to a flattening of hierarchies and an increase of uncertainties. Communication management was seen as the main area for improvement. Physiotherapists hoped that their involvement would improve recognition of the profession but feared that working conditions would deteriorate after the crisis.

**Conclusions:**

The physiotherapists expressed high dedication to their profession and pride to be part of the “war effort” during the crisis. The stress level was partly tempered by the solidarity amongst health professionals and distraction by engaging in action. Despite the mental load, this situation was also seen as an opportunity to grow at a personal and professional level. The healthcare system capacity having not been exceeded in Switzerland, less distress related to death and powerlessness were expressed than in other studies investigating healthcare professionals’ experience of the COVID-19 crisis.

**Supplementary Information:**

The online version contains supplementary material available at 10.1186/s40945-023-00169-2.



## Introduction

The World Health Organization was informed on 31 December 2019 that a case of pneumonia of unknown cause, subsequently identified as originating from the SARS-CoV-2 virus, had been detected in the city of Wuhan in Hubei Chinese province [1]. The first case of the Coronavirus disease (COVID-19) caused by this virus outside of China was confirmed on 13 January 2020. Since then, the disease has spread worldwide. The presence of the coronavirus SARS-CoV-2 has been documented in Switzerland for the first time on 24 February 2020 [2]. From this point and considering the pressure on the health system caused by the increases of cases in the border countries, in northern Italy initially, hospitals activated the health plans for pandemics.

As a result, a reassignment of professionals was organised within the various departments of hospitals. The capacity of intensive care beds throughout Switzerland was rapidly increased from 240 in normal times to 395, which revealed sufficient to prevent hospitals saturation during the COVID-19 first wave until June 2020 [3, 4]. More specifically in the university hospital in which this research was conducted, the capacity increased from 35 to 83 intensive care beds, while a peak of 63 patients was reached during the first wave (41 patients with and 22 patients without COVID-19).

Although Swiss healthcare professionals were not confronted to triage for intensive-care treatment caused by resource scarcity, they nevertheless had to face a variety of complex challenges, including the confrontation to a new potentially lethal communicable disease and a complete reorganisation of their working environment. Numerous publications have addressed the subjective experience of health professionals in general, but publications addressing the subjective experience of physiotherapists working in acute care are less frequent and originate from diverse contexts in terms of country, health system and impact of the pandemic [5–9]. These publications concern countries (Spain, USA, Nigeria) where the health situation was more critical than in Switzerland [10]. It is therefore of interest to carry out research specifically on the experiences of Swiss physiotherapists in order to identify common features and differences according to professions and general context during the outbreak.

Learning more about the physiotherapists’ experience, perceived role and perception of events during the crisis, as well as their recovery and projection into the post-crisis future may be useful to inform stakeholders at various levels. From a social and professional perspective, it may document the subjective experience of physiotherapists during this unprecedented crisis and be revealing of the professional values underlying their actions. From a managerial point of view, it can provide a useful assessment, point out adjustments to be made for future crises and highlight some expectations of physiotherapists in terms of management. From a psycho-social perspective, it may document in which way physiotherapists felt affected and recovered from the situation.

Thus, the objective of this qualitative study was to investigate the experience of physiotherapists working in a university hospital during the 1^st^ wave of the COVID-19 crisis, more specifically with respect to their subjective experience, professional involvement, perception of management and implications for the future.

## Methods

### Design

A qualitative study using an interpretative approach was conducted by crossing the views of a physiotherapist and an anthropologist, both accustomed to studying the context of physiotherapists' work in the hospital [11], at the request of the hospital Physiotherapy Board. This study is qualified as interpretative because it seeks to report subjective descriptions complemented by interpretations anchored in the participants' accounts and concepts discussed in the literature. Focus groups were chosen as method, for their suitability to efficiently collect a consistent amount of subjective data from a population, and to identify points of consensus or of debate within a group [12, 13]. Consolidated criteria for reporting qualitative studies (COREQ) toolbox were used for study reporting [14].

### Ethics

The local research ethics committee certified that the project could be carried out in accordance with Swiss ethical regulations without requiring an in-depth assessment from them. Still, the recruitment process was designed so that no pressure could be exerted on the participants regarding their participation and measures were taken to guarantee the anonymity, confidentially and the safety of data storage. Potential participants were given information and consent forms. Participation in the study was conditional on signing the consent form.

### Context

The study was conducted in CHUV (Centre Hospitalier Universitaire Vaudois), a Swiss acute care university hospital, following the COVID-19 first wave. The focus groups were held in a quiet room in the hospital, away from the participants' departments, reserved for the purpose of the study.

### Participants

A message was sent by the internal Physiotherapy Board on 19 May 2020 to all physiotherapists working in the university hospital. Volunteers were asked to submit their applications directly to the investigator (CP) and to arrange their release for the interviews on 8 or 10 June via their direct hierarchy, so that their anonymity was preserved towards their higher hierarchy including members of the Physiotherapy Board. Each volunteer received a detailed information form, as well as a consent form to be returned to the investigator prior to the interviews.

A purposeful sampling method was planned [15], to set up 2 interview groups of at least 5 physiotherapists with different characteristics in terms of socio-demography, professional profile and involvement in the COVID crisis. It was estimated that two focus groups would be necessary to contrast the groups' perspectives in case they differed. As the recommended group size is five to eight [12], it was estimated that groups of at least 5 participants should be constituted. It was also estimated that a sample of at least 10 participants would be sufficient to reflect the diversity of profiles present in the hospital according to the following four criteria: gender, age, departments, exposure to patients with COVID. All physiotherapists working in the institution could be included, to the exception of those having a managerial function. No other criteria were set, as the study aimed to reflect the experience of physiotherapy staff and not just those who had direct contact with COVID-19-infected patients.

### Research team and reflexivity

The investigator was an advanced practice physiotherapist in the musculoskeletal department of the hospital and an associate professor in physiotherapy in a university. He holds a PhD in health sciences with research experience in qualitative research. He was not involved in patients’ care during the COVID-19 crisis and thus was not directly affected by the hospital reorganisations. He therefore had an in-depth knowledge of the field of investigation while having a position of external observer. The researcher was a colleague of the participants, with no hierarchical relationship to them.

### Data collection

An interview guide was developed by the investigator (CP) to conduct semi-directive focus group interviews (Additional file 1: Appendix 1). The dimensions addressed in the guide were: General impression, Professional involvement, Crisis management, Feelings, Future perspectives and Other impressions. The interview guide was analysed and approved by the Physiotherapy Board to ensure its relevance and completeness. The focus group meetings took place in the hospital, outside the physiotherapy department, and lasted 2 h each. They were led by the investigator (CP), who acted as a neutral moderator, accompanied by an administrative staff member who took notes of turns to speak, managed the recording and acted as a timekeeper. The interviews were saved on two voice recorders to prevent loss in the event of technical problems. 

### Data analysis

The interviews were transcribed in full by the administrative staff member for analysis. They were then entered into the MAXQDA qualitative analysis software version 2020, in which the investigator had previously developed a predefined coding system based on the interview guide dimensions. No emergent themes were derived from the interviews, although this possibility was left open in the methodological approach. The participant discourses were coded by the investigator using 18 codes and 2 subcodes related to the previously mentioned six dimensions investigated in the interviews (Additional file 1: Appendix 2) [16]. Quotations were then extracted for each code, and themes were defined using inductive thematic analysis [17]. The analysis was flexible and iterative [18]. Themes were developed by attempting to identify recurring patterns or themes across the coded segments in order to capture the essence of the data and provide a framework for analysis. The themes were then refined or merged as necessary during the analysis process. Themes were then reviewed for adequation and completeness and final naming was made to illustrate the expressed experiences of the participants [19].

### Rigour

Member checking was implemented to ensure the credibility of the study [20]. The research report was sent to the participants, to ensure the reliability and completeness of the interpretation. Ten participants indicated their explicit approval without modification, and two did not respond following two recall messages at 15 days interval, which was considered as tacit approval. The study was audited by a health anthropologist with experience in interdisciplinary research with physiotherapists (RF, female ordinary professor, PhD in human sciences), to ensure dependability and confirmability [20] and provide an external social science perspective on the analyses. She discussed the methodological approach and the development of the interview guide with the researcher before the start of the study. During the analysis, she checked the analysis method (how the codes were used to identify relevant quotations and how the themes were derived from the data and named), read the first analysis draft, discussed results, checked the relevance of the interpretations in terms of trustworthiness to citations and social theories and supplemented the interpretations and constant hypotheses process. The characteristics of the respondents are not reported with their quotes cited in the Results section to preserve their anonymity.

## Results

### Study sample

The planned purposeful sampling method was implemented in order to set up 2 interview groups of 5 physiotherapists [21]. As 13 physiotherapists out of a workforce of 170 volunteered, among which one was excluded due to exclusion criteria (managerial function), 12 were finally accepted, so that no eligible volunteer was excluded. As the expected diversity in terms of gender, age, departments and exposure to patients with COVID was achieved, no additional participants were requested. Thus, 2 groups of 6 people were formed, accounting for the expected variety of profiles within a group and the availability of the participants (Table 1).

**Table 1:** Characteristics of the participants

Characteristics of the participants		
Sex (Male/Female)	6/6	
Department	Cardio-respiratory	4
	Musculoskeletal	4
	Mother and children	2
	Internal medicine	2
Affectation in the crisis	1^st^ front in his/her regular affectation^a^	4
	Transferred to 1^st^ front	4
	Transferred to 2^nd^ front^b^	3
	Non COVID department	1
Function	Physiotherapist	12
Mutation during the crisis	Yes	7
	No	5
Age (median; 1^st^ quartile; 3rd quartile; min; max)	32; 28; 36; 26; 40	
Years of experience (median; 1^st^ quartile; 3rd quartile;; min; max)	8; 5; 10; 2; 15	
Years working for this employer (median; 1^st^ quartile; 3rd quartile; min; max)	7; 5; 9; 1; 15	

^a^1^st^ front: treatment of patient with COVID-19

^b^2^nd^ front: replacement of colleagues called to treat patients with COVID-19

### Motivations to participate and general impressions: participating in a “war effort” or being left out

The reasons to volunteer for the focus groups were the wish to share experiences, express feelings about the crisis period and contribute to drawing lessons from it to improve the functioning of the institution. PT 3 said:” Why* I came [to the focus group], because […] I think it was an exceptional period, exceptional issues came out that can indeed, I think that it can help physiotherapy in general, both on the recognition of competences and on two or three things of that kind”.*

Those directly involved in the crisis felt that they were participating in a "war effort", as expressed by PT 10: “*You could see the services being emptied little by little and I wanted to be useful and to participate in the war effort if it was possible to do so”.* Simultaneously, those whose department had been emptied in anticipation of the admission of patients with COVID-19 felt anxious and idle because of the reduced activity, like expressed by PT 8 “*We did nothing, we just waited for it to come and were there, we waited, we waited because we'd been told the peak is coming, it was going to happen. It was horrible, this waiting doing nothing”* and PT 4 “*I had the same feeling uh… indeed this waiting that increased the anxiety of wondering what was going to happen… and that's why I asked to be transferred because I couldn't wait any longer […] once we were in the heat of the action, well, then it was better.”*

### Professional involvement while having little clinical knowledge and distance

Participants expressed a high level of commitment to their duty during the crisis, despite the potentially serious repercussions in the event of contamination, like mentioned by PT 9: “(*…) the idea is war, we have to help, we're going there”,* PT3 *“It's your duty to be there and do what you can do”* and PT 8 *“We were all on the front line risking our lives… so it's not like that in the end… but at the beginning when it happened, it was as if the COVID was going to kill half the people…”.*

Physiotherapists had to adapt to an unprecedented situation and in a context in which best practice recommendations were insufficient. Thus, the work had to be carried out in uncertainty like pointed out by PT 11 “*I have the impression that we were so flexible that regarding the information we didn't have, we said OK, never mind… we'll fix something, we'll do it like that,* [*without the information*]. Looking backward, some wondered to what extent their interventions were adapted to their patients: “*Sometimes I wondered if I was the one who had made the patient worse?* (PT7). However, participants felt that it was very important that their work be done.

Some mutations to another department were made under the pressure of emergency. In those circumstances, the specific roles of each collaborator were perceived as insufficiently precise. Should a comparable situation occur again, expectations toward those who were received into a new service and those who were to train them were perceived as important to define precisely.

Physiotherapists demonstrated a high degree of adaptability to fulfil their mission but were concerned that this adaptability gave the impression that staff members are interchangeable, which could jeopardise the recognition of their expert skills: “O*ne of the problems with this crisis is that we have shown that we can do many things and therefore we have shown that we are also interchangeable*” (PT3).

### Contrasting feelings of solidarity, capability and mental burden

Exchanges of staff between physiotherapy departments and working towards a common goal reinforced the perception of a physiotherapy team entity, whereas direct attachment to one's department usually prevails. On solidarity, PT11 said “*This COVID period really made it possible to federate the physiotherapeutic entity within the hospital”,* PT9 *“I'm quite in agreement with the fact of solidarity as well … something that struck me a lot is the trust of almost everyone, I had the impression that we were really trusted”,* and PT 6 added *“This solidarity we have all the time… it's just that it's been exacerbated… we have it all the time…”.* Being able to rely on each other, in the absence of hierarchy or judgement between colleagues, stood out strongly and was appreciated. Intra- and inter-professional collaboration developed, which facilitated interprofessional collaboration, like pointed out by PT 11:*”* *We* *were all a bit scared of the same thing and so we really had to face this issue together, and it really helped to make things much more fluid in interprofessional and interdisciplinary interactions”*. Living this solidarity was seen as a strong and rewarding experience.

The crisis also involved a mental burden, resulting from issues within and outside the hospital. Internally, sources of mental burden were the overabundance of information, constant adaptation, need for flexibility, projection into the unknown, need for initiative, need for cross-training and potential risk to self. Externally, the mental burden arose from managing life outside the hospital (family, child education, etc.) and the anxious global atmosphere (relatives, discussions, media, etc.), as expressed by PT 6: “*You go home, you have to watch the news, what's going on in Italy, so you're in it again, you come back here, you don't have any holidays, you leave… well, at some point, it's also that we're having a hard time because our society is like that and there are so many things on social networks, I think”*. The hospital was sometimes seen as a refuge, compared to the outside world where the propagation of the virus was less controlled, as pointed out by PT 8: “*We were basically told, you risk your lives at work […] this is a kind of big house, in the beginning it was, at least for me, almost more reassuring to be at work than to be outside of work”*.

The workload was very heavy in COVID-19 services, due to the number of patients and the ongoing reorganisation but was not perceived as insurmountable. Nevertheless, the participants expressed that fatigue would have been harder to manage in the event of a longer-lasting crisis (without knowing at the time that more waves were to come), as expressed by PT 10: “*Maybe if the situation had lasted a year, it would perhaps have led to other problems, precisely of flexibility among people who were very flexible at the beginning, who after a while would have said: enough, stop it's a little too much”*.

Regarding service transfers, volunteering was an important factor in containing stress, as expressed by PT 10: “*If people had been forced to change departments, the stress factor would have been much greater. We decided to do it anyway. There is always a little bit of stress from the unknown but in the end, we wanted to do it”*. The preparation of transfers was also important to contain the stress of the mutated physiotherapists and of those receiving them.

Concerning the access to protection material, physiotherapists perceived that their situation was fragile. As they usually work on several units simultaneously, they were not considered as fully-fledged staff members of a service and thus didn’t have priority in receiving supplies, as mentioned by PT 4: “*The physiotherapist is a little bit the consultant in the service […]. So, the service has its own material management, and you can only take what is made available to you, and if there's nothing, well… you can't take it.”*

Views on the crisis varied from a positive and formative experience to a difficult experience of unrecognised investment. On the positive side, physiotherapists expressed pride in the duty they had accomplished, solidarity and a certain power gained in the health care field, which they would like to see continue. Some had lived a common experience, which appeared as a transformation or major event in their lives. PT 12 had this perception: “*I can really see that it has brought me a lot of things at last… on a professional level or on a global level and I think that at last… it's changed I think everyone or well… not everyone but well… I'm getting a lot of positive things from it.”*

On the negative side, the mental burden that impacted both professional and personal aspects of life left little space for recuperation, like pointed out by PT 6 *“We were in the stress of not knowing what was happening to us, […] I didn't take it as rewarding, in retrospect perhaps, but I found it heavy, really very heavy and we hadn’t got into the hard and fast stuff… yeah, there you go… Rather tiring and also not recognised [..] at the beginning we had a bit of a hard time and then when things stabilised, we could ease off the pressure and say: well, people are competent, they manage it, and then, we were swimming in the mass of patients, the mass of work, the mass of health professionals, well… for me I found it quite tiring…*”.

### Crisis management, communication challenges and temporary increase in power of the field

The reorganisation of the hospital was seen as impressive, and participants acknowledged that the system in place had proved resilient, like illustrated by PT 11 “*What surprised me was the increase in power in the services, well… intensive care medicine or with all this fighting activity where clearly, we increased from 5 to 6 units, 7 units, 8 units, 9 units finally… it's really, almost exponential and I thought it was quite impressive… yeah really, this increase in power”*. In parallel to the established power, a power from the field was temporarily set up to respond to emergencies in care situations, like expressed by PT 3: “*There was this power of the field which was quite pleasant in the end, and which we are now seeing disappearing very quickly”*. There was a consensus that communication was the main area for improvement, like felt by PT 11 “*I think that in moments of crisis, communication is an essential point and I think that there were really, really shortcomings there”*.

The emergency reorganisation considerably disrupted communication and exchanges between staff members, as they did not know each other. So, autonomy in initiative became essential. This was appreciated but was considered as suboptimal for the homogeneity of practices and rigour in care.

Physiotherapists felt overwhelmed by information and rapid changes in procedures they had to stay up to date with: “*We also had a flow of information that was extraordinary. I’m thinking about the e-mails, […] all the changes in procedures and so on… yeah, we already had to follow the rhythm and then, … We were off for 2 days, we had received so many emails, so we had to get back up to date”* (PT 11). Physiotherapists attached great importance to reliable information, a potentially anxiety-provoking communication being considered as preferable to a softened communication in order to contain anxiety. On this issue, information about the protection material was perceived as truncated at the beginning of the crisis when uncertainties prevailed as to wear the mask. Moreover, the cancellation of team meetings due to the sanitary precautions limited the opportunities to discuss the implications of information.

Physiotherapists valued direct contact with a field manager, for operational and clinical aspects, as well as to make information interpretable, as positively experienced by PT4: “*He is a field manager, which means that he understands the work that needs to be done. He is present and if he has to replace me, he is able to do it. And if I express a concern or a problem to him, he understands it because he knows how to put himself in my place”.* The role of physiotherapy department managers, who are not involved in patient care, was less visible and less easily circumscribed by the physiotherapists. The public valorisation of physiotherapy in the public media as an active profession in the crisis effort was also perceived as insufficient.

In a context that implies personal commitment, administrative issues concerning time compensation and holidays were very negatively perceived. Harmonisation and equity were perceived as fundamental in administrative management.

### Looking ahead, areas for improvement and fear of the impact of the crisis in the longer term

Many participants wished to use the experience gained to improve the day-to-day functioning of the hospital, as illustrated by PT 7: “*I think we have to, we have to, we have to keep this and continue to… because we quickly have barriers, we can't change services like that […] I think we have to try to get something out of it and tell ourselves that we have to be more polyvalent…”*. They presented themselves as ready to face a repeated crisis but hoped that the lessons from the current crisis would be drawn. Some wished the reactivity and autonomy, as well as the decompartmentalisation between departments to be maintained.

A mental burden by anticipation was expressed regarding the possibility of a 2^nd^ COVID wave —at the time of the interview considered a mere hypothesis— like expressed by PT 9: “*If there were to be a second wave in September or October or I don't know, next year it might be a bit harder to start again and get back into the process”*. It was suggested that sets of physiotherapy specifications and skills inventories should be more precisely defined beforehand to help manage staff reassignments.

The exchanges strengthened the feeling that skills need to be shared between physiotherapists from different services. However, the creation of a pool of physiotherapists with generic skills was clearly unwanted. After having shown flexibility and adaptability, physiotherapists feared to be considered as interchangeable across departments, regardless of their skills.

They hoped for a lasting and concrete recognition process, while being pessimistic about its realisation, as the costs induced by the crisis may put the hospital under pressure and finally have repercussions on staff. This was expressed by PT 3: “*For the moment we are applauded but next year I'm not sure. Already the budgets that are announced for training courses and other things are going to be horrible. And the budget for the workplaces, they are going to cut it […] I'm very, very afraid of that, I confess”.*

In line with expressed perception of the current crisis, the areas for future change were anticipation of a 2^nd^ wave, improvement of communication, harmonisation of administrative practices, sharing of skills between services, drawing up physiotherapy specifications, concrete recognition of professionals and accessibility to protection equipment.

## Discussion

This study investigated the experience of physiotherapists during the 1^st^ wave of the COVID-19 crisis in a Swiss university hospital, with respect to their subjective experience, professional involvement, perception of management and perceived implications for the future. Thus, the discussion of the results was structured according to these four categories.

### A powerful experience that revealed core values

The general impressions of physiotherapist show that the 1^st^ wave of the COVID-19 was an intense experience that revealed the involvement and core values of stakeholders in the hospital, due to the need to quickly shape a new functioning. Warlike terms were frequently used to describe the situation. This disruption of the routine was seen as an opportunity to learn lessons for future improvements. Studies conducted in other health professions and cultural contexts also found that the crisis could be seen as an opportunity for growing at the personal and professional level [22, 23].

The most prevalent negative and positive issues were related to the increased exposure to stressful situations and to the solidarity that appeared as an efficient defence mechanism to affront the shared exposition to stress. Similar feelings were seen in other healthcare teams [24, 25]. However, this solidarity appeared as fragile when the interests of different professional groups entered in competition, like during the period of protection material shortages, which was also stated elsewhere [7, 9, 26, 27]. In contrast to some other studies, there was no mention of stigmatisation or avoidance by others in the interviews [5, 28]. The position of physiotherapists was perceived as delicate because their function was not dedicated to a unique service of which they are considered as fully-fledged members. So, if solidarity was felt, dissensions between professional groups could also emerge.

### A wide variety of stress perceptions

The determination to act and to fulfil their duty, even at the risk of serious personal consequences like contamination and burnout, was also striking. The involvement against the outbreak was compared to a war effort, similarly to another study conducted in Spain [8]. Such determination seems to have been common in health workers and to be a source of pride [5, 8, 9, 23–25, 29–32]. This altruism is a defence mechanism against stress that has also been demonstrated efficient in containing the stress in other health workers during the crisis [23, 33]. The impossibility to rely on this mechanism, coupled to powerlessness from being deprived from action means, may explain why those who were idle expressed the highest anxiety. As studies during the outbreak mainly investigated the situation of health professionals directly involved in the management of patients with COVID, they did not highlight the importance of the psychological burden for those who were deprived of activity due to the suspension of the usual activities of their department. Nevertheless, there were significant sources of stress to manage for physiotherapists, relative to the exposure to danger, the uncertainty about protection measures for self and patients, the information management, the responsibilities toward patients and less experienced colleagues, and the instant adaptation to a radically changed environment. Those who could take personal distance when leaving their workplace seemed to cope better than those who couldn’t, due to the repeated recall of the situation by relatives and media. The latter could not rely on distraction, a helpful coping strategy in situations of low control [34, 35]. Other studies also reported the important role played by the family and friends on the stress of healthcare workers during the crisis [24].

Unsurprisingly, the stress sources were essentially related to the critical health situation and the working conditions. Conversely to some countries, the Swiss health system having been stressed but not overwhelmed, the physiotherapists of the hospital were not confronted to moral and ethical challenges related to prioritisation of care according to available resources. This may explain why the reported stress was lower in this study than in many others, and why the distress related to the confrontation to death was not expressed openly during the interviews contrary to many publications [6, 9, 32, 36–39].

### Crisis management and recognition expectations

The need for immediacy in decision-making and action led to a flattening of hierarchies. Confidence seems to have been given to those who felt able to take on the patients’ care, and targeted support to the less experienced ones. The recognition of everyone's common effort seems to have been an overriding principle that eroded hierarchical or corporatist conceptions of labour relations. Other studies reported controversial results on hierarchical issues, one showing also a valued increase in autonomy [25], another finding an exacerbation of hierarchical inequalities [30].

While this change in professional relationships was seen as rewarding, it also raised questions about the weakening of the measures that ensure safety and adequacy of care. In the absence of professional recommendations at this stage of the crisis, decisions were taken based on experience and clinical reasoning, without possible reliance on evidence-based knowledge, a problem that also affected other professions [26]. This increased the physiotherapists’ responsibility concerning clinical decisions and left them unsure concerning the relevance of some interventions, which may have induced complicated ethical challenges for them. This statement highlighted the need for training to adequately address the ethical issues that may arise in complex acute care situations [6]. The importance of field managers who can share their clinical experience and provide guidance in uncertain circumstances was valued, like is the case in nurses [29].

Concerning the crisis management, the physiotherapists unanimously recognised that the main issue i.e., the hospital complete reorganisation to face the arrival of a wave of patients, was successful. Nevertheless, they critically analysed some aspects of the management, like structuration and selection of communication, anticipation of unavailability of managers and clarification of the roles of physiotherapists in crisis situations.

Physiotherapists strongly expected that the information delivered was trustworthy. The expression of uncertainties by the communicators was said to be preferable to retention of information, even with the intention to avoid anxiety. The issue of the dissemination of information was also stated in another publication [40]. Concrete recognition of involvement was also highly expected. Though their participation to the common effort was not negotiated in the heat of the crisis, a posteriori recognition by administrative measures, esteem, wage compensations and enhancement seemed to be expected in return.

### Projection into the future and tensions regarding interprofessionality

The quotes about the projection into the near future showed a relief after the end of the COVID-19 first wave and the expectation that the return to normality would be enriched by the acquired experience. This implied to address the identified shortcomings and to draw lessons from the positive experiences, which showed that efficient alternatives to the usual functioning were possible. Since the data collection of this study, several waves of COVID-19 occurred. Adjustment to crisis management practices of the hospital were seen concerning the reduction of the flow of information, the possible reliance on evidence-based guidelines and the availability of protection material, in a more stable context.

There was a tension in the discourse about interprofessionality, between the importance of being part of the caring community and the risk of losing recognition of specific professional skills and being reduced to a generic role of health professional.

There was also a tension between hopes and fears concerning future evolutions. On one hand, it was hoped that the demonstration of dedication of physiotherapists to their professional duty would bring moral and concrete social recognition. On the other hand, it was feared that the previous operational mode would simply return, though with more limited resources.

### Study limitations

The results are based on a monocentric data collection. The characteristics of the hospital and local conditions should therefore be taken into account before transferring the results to other settings. The objective of this qualitative research was to highlight the elements that shaped the experience of physiotherapists during the 1^st^ wave of COVID rather than to produce generalizable results.

No double checking of analysis could be implemented. The possible interpretation bias related to the researcher subjectivity was contained by requiring the approval of the results by the participants, and the detailed results were discussed with a health anthropologist to ensure dependability and confirmability [20] and provide a perspective from outside the profession [41].

A purposeful sampling was planned. As the expected variety of profiles could be reached with the sample of the 12 first physiotherapists who volunteered no additional participant was requested (Table 1). Only two focus groups were conducted, which is below recommendations for qualitative research and may not be sufficient to fully reflect the diversity of physiotherapists' experiences [42]. However, the purpose of the study was to reflect the specific experiences within the context of the hospital only. Therefore, given the number of departments in the hospital, it was estimated that two focus groups of 6 participants would be sufficient to reflect the variety of perspectives according to the possible physiotherapy positions within the hospital and to compare the data between the two groups. The number of participants is in line with common practice in qualitative research [43]. As the experiences reported within the two focus groups converged, increasing their number would have had marginal added value to the results. The use of focus groups may have favoured the expression of hospital functioning issues at the expense of more personal matters, as the expression of personal experiences may be modified or inhibited by group effects [12, 44]. However, this was a limited drawback in the context of this study, which explored professional but not intimate experiences. On the other hand, focus groups can also have been beneficial in stimulating discussion and opening up new perspectives for participants [7].

### Further investigations

The COVID-19 crisis had not been completely resolved at the time of the interviews. Further research would be useful to understand how the physiotherapists experienced the long-lasting COVID-19 crisis over time. As this study did not enrol managers, it would be useful for the profession to also investigate their experience to contrast views.

## Conclusion

This qualitative research provided an in-depth analysis of the physiotherapists experience in a university hospital during the COVID-19 first wave in Switzerland. The physiotherapists expressed high dedication to their profession and pride to be part of the “war effort” during the crisis. They also expressed a high stress level related to workload, information flow and uncertainties, which was partly tempered by the solidarity amongst health professionals and the distracting from stress by engaging in action, for those would were directly involved in the treatment of patients with COVID-19. Despite the mental burden, this crisis was also seen as an opportunity to draw lessons based on the full-scale experiences made during this unprecedented hospital reorganisation. This experience raised hopes for better recognition of the profession and simultaneously fears of lack of recognition and the negative impact of the crisis on health care resources.

Future research should investigate the evolution of the physiotherapists’ experience in the following phases of the crisis to understand how they adapted on a longer term. The perception of managers would also be of interest to provide a more diversified view of the crisis experience.

### Supplementary Information

**Additional file 1.**

## Data Availability

The data are not made available in order to preserve the confidentiality of respondents.
