# Supplementary material for: In-between duty and hope for recognition, the experience of physiotherapists working in a university hospital during the COVID-19 first wave in Switzerland: a qualitative study based on focus groups
Source: Arch Physiother. 2023 Aug 17;13:16. doi: 10.1186/s40945-023-00169-2 (PMC10436384; doi:10.1186/s40945-023-00169-2)
Supplement: Supplementary file 1 — Additional file 1. [file 40945_2023_169_MOESM1_ESM.docx]

# **Appendices**

Appendix 1

## **Interview guide**

**Introduction**

Reminder of the objective and the course of the focus group interview:

Objective: To find out what affected the physiotherapists who were exposed to a major change in their professional activities in a context of reorganisation of care in a crisis situation.

Course:

- Introduce the investigator-moderator, his role, why he is in charge of this focus: to explain the role of moderator
- Introduce the research collaborator, her role: keeping the framework, keeping time, taking notes, 2nd glance
- Explanation of how a focus group is conducted: methods, usefulness, role of the moderator and timekeeper, confidentiality and anonymity, transmission to the hierarchy, specify the themes to be investigated, explanation of the choice of selection.
- Importance of confidentiality towards the hierarchy and between colleagues. Opinions may differ, express them with respect

**Questions**

**Introduction of participants**

Can you briefly introduce yourself and say what motivated you to take part in this survey?

**General impressions**

You have just been through a very special time, what has affected you the most in what has just happened?

**Professional involvement**

What did you think of the adaptations you were asked to make?

- Flexibility
- Change of services
- Social distancing
- Review/refine training
- Cancellation of treatments
- Adequacy of the role you have been allocated

Do you feel you have been able to fulfil your role as a physiotherapist as you see it?

- Taking care of patients
- Implementation of physiotherapy techniques
- Relationship with the patient, humanity in care,

Does this make you rethink your role as a physiotherapist in any way?

How do you feel about the working relationships during this period?

- Collaboration during care
- Colleagues' attitude towards each other
- Colleague support and mutual aid
- Interaction with the hierarchy
- More broadly, how did you feel about communication between professions and departments?

**Crisis management**

What do you think of the way the situation was handled in your teams?

- Competence in crisis management
- Quality of the information transmitted
- Way of transmitting information (institutional and within the team)
- Way of communicating and taking into account the opinions of teams/people
- Administrative management: schedules, holidays, social distancing

**Feelings**

How do you currently feel about what you experienced during the crisis?

- Overall positive or negative?
  - Feeling of emptiness? exhaustion? dynamism?
  - Feeling of self-worth or worthlessness?
  - Feeling of sacrifice or recognition?

What made you feel good, or on the contrary, weakened you or was difficult to live with?

- Were there hard moments?
- Were there any decisions that displeased or disappointed you?

What were the stress factors in the situation for you?

- Signs of stress (anxiety sleep nervousness ruminations ...)
- Has the ability to separate professional and personal life been affected?
- Did you feel your health was threatened?
- Fear of infecting others?
- Fear of not being able to cope if cases spill over?
- Difficulty adapting to change and uncertainty?

**Projection into the future**

The period of containment and de-containment brought about many changes and a new experience: what aspects would you like to see continue, or on the contrary end?

- How do you see the future? What changes do you want to see continue?
- Would you like to return to the situation before the crisis, or not quite?
- What would you like to take back from the old and new world?
- What aspects of the normal situation were you happy to see disappear?

What will we have to pay attention to in the future?

- In the organisation?
- In the care of the patients?
- In the communication?

What if it lasts, e.g. in case of a second wave, what changes should be maintained and what should return to normal in the near future?

- How far would the current crisis situation be acceptable in the long term?
- What aspects of the normal situation did you miss and do you miss?
- What would you like to return to quickly if possible?
- And if a similar or different crisis happens again, what would you need to do the same or change?

Has the crisis made you change your view of your role and that of physiotherapy, in what way?

- About your daily work?
- About your role in the team?
- About the role of the team in the hospital?
- About the role of physiotherapy in the health care system

**Other impressions**

Anything else you would like to say, any message you would like to pass on?

Appendix 2

## **List of codes**

| **THEMES** | **CODES** |
| --- | --- |
| **General impression** | Motivation to participate in the focus group  Milestone event  Activity during the crisis |
| **Crisis management** | Perception of information  Perception of communication  Perception of the organisation  Perception of management  Perception of the attitude of the hierarchy |
| **Professional involvement** | Motivation to act in the crisis  Role to be assumed  Personal investment  Problems in patient care  Professional competence |
| **Feelings** | Perception of collaboration at physiotherapy level  Perception of collaboration at hospital level  Mental load  Personal feeling  Working relationships |
| **Projection into the future** | Projection into the future |
| **Other impressions** | Message to be passed on |
